# High Serum Ferritin Levels among Blood Transfused Thalassemic Patients Admitted to the Department of Paediatrics in a Tertiary Care Centre: A Descriptive Cross-Sectional Study

**DOI:** 10.31729/jnma.8195

**Published:** 2023-06-30

**Authors:** Damodar Tiwari, Sweta Kumari Gupta, Narayan Bahadur Thapa, Kiran Devkota

**Affiliations:** 1 Department of Paediatrics, Bharatpur Hospital, Bharatpur, Chitwan, Nepal

**Keywords:** *blood transfusion*, *ferritin*, *thalassemia*

## Abstract

**Introduction:**

Raised serum ferritin levels often indicate iron overload, but they are not specific as the levels are elevated in inflammatory disorders, liver diseases, alcohol excess, or malignancy. If regular transfusions are required for the patient with thalassemia, this doubles the rate of iron accumulation leading to earlier massive iron overload and iron-related damage. The aim of this study aimed to find out the prevalence of high serum ferritin levels among blood-transfused thalassemic patients admitted to the Department of Paediatrics in a tertiary care centre.

**Methods:**

A descriptive cross-sectional study was conducted at a tertiary care centre from 1 March 2022 to 31 December 2022. Ethical approval was taken from the Institutional Review Committee (Reference number: 078/79-017/HG). Children who were confirmed by haemoglobin electrophoresis on regular blood transfusion were included in the study. Those who did not gave consent were excluded from the study. Convenience sampling method was used. Point estimate and 90% Confidence Interval were calculated.

**Results:**

Out of 53 cases, the prevalence of high serum ferritin level was seen in 46 (88.79%) (80.3097.28, 95% Confidence Interval). Among 46, 34 (73.91%) had serum ferritin levels of more than 1000 to 2500 ng/ml whereas 12 (26.09%) had more than 25000 ng/ml.

**Conclusions:**

The prevalence of high serum ferritin levels among blood transfused thalassemic patients admitted to the Department of Paediatrics in a tertiary care centre was found to be higher than in other studies done in similar settings.

## INTRODUCTION

Raised serum ferritin levels often indicate iron overload, but they are not specific as the levels are elevated in inflammatory disorders, liver disease, alcohol excess, or malignancy. Blood transfusions in people with thalassemia can result in iron excess, which raises serum ferritin levels.^1^

If regular transfusions are required for the patient with thalassemia, this doubles the rate of iron accumulation leading to earlier massive iron overload and iron-related damage.^2^ The carrier of the thalassemia gene is higher in the Tharu community among the Nepalese population.^3^ Young children with Beta thalassemia usually become symptomatic during the first year of life.^4^

The aim of this study was to find out the prevalence of high serum ferritin levels among blood-transfused thalassemic patients admitted to the Department of Paediatrics in a tertiary care centre.

## METHODS

This descriptive cross-sectional study was conducted at Bharatpur Hospital, Bharatpur, Chitwan from 1 March 2022 to 31 December 2022. Ethical approval was taken from the Institutional Review Committee of the Bharatpur Hospital (Reference number: 078/79-017/HG). Children who were confirmed by haemoglobin electrophoresis on regular blood transfusion were included in the study. Those who did not gave consent were excluded from the study. Convenience sampling method was used. The sample size was calculated using the following formula:


$$\begin{array}{ll} \text{n} & ={\text{Z}}^{2}\times \frac{\text{p}\times \text{q}}{{\text{e}}^{\text{2}}}\\ & =1.64^{2}\times \frac{0.50\times 0.50}{0.115^{2}}\\ & =51 \end{array}$$

Where,

n = minimum required sample size
Z = 1.96 at 90% Confidence Interval (CI)
p = prevalence taken as 50% for maximum sample size calculation
q = 1-p
e = margin of error, 11.5%

The minimum required sample size was 51. However, the final sample size taken was 53.

The methods were explained to the parents and older children with written consent. The clinical details including the sex of the children, age of the children at diagnosis, and caste of the children were recorded. Blood samples from each patient were collected with the aseptic method and sent for estimation of serum ferritin level. A target serum ferritin level in transfusion-dependent thalassemia is approximately 1000 ng/ml is the standard practice (Thalassemia International Federation guideline 2000).^5^

Data were entered and analysed using IBM SPSS Statistics version 20.0. Point estimate and 90% CI were calculated.

## RESULTS

Out of 53 cases, the prevalence of high serum ferritin level was seen in 46 (88.79%) (80.30-97.28, 95% CI). Among 46, 34 (73.91%) had serum ferritin levels of more than 1000 to 2500 ng/ml and 12 (26.09%) had more than 2500 ng/ml (Table 1).

**Table 1:** Serum ferritin level in range among patients with high serum ferritin level (n= 46).

Range	n (%)
More than 1000 to 2500	34 (73.91)
More than 2500	12 (26.09)

**Figure 1:**
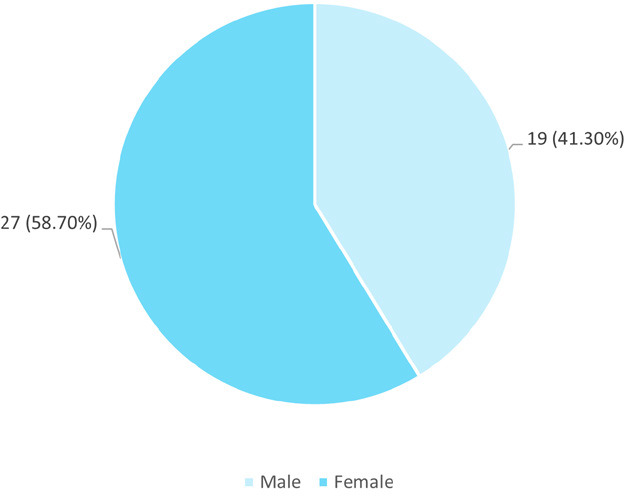
Gender distribution among patients with high serum ferritin levels (n= 46).

Among 46 patients, 27 (58.70%) were male while 19 (41.30%) were female (Figure 1).

The mean age was 8.66+4.064 years. Majority of patients 21 (45.65%) with high serum ferritin levels belonged to the age group of 10 to 14 years (Table 2).

**Table 2:** Age distribution among patients with high serum ferritin levels (n= 46).

Age (years)	n (%)
Under 5	11 (23.91)
5 to 9	14 (30.43)
10 to 14	21 (45.65)

High serum ferritin level was mostly seen among Tharu/Chaudhary 26 (56.52%) (Table 3).

**Table 3:** Caste distribution (n= 46).

Caste	n (%)
Tharu/Chaudhary	26 (56.52)
Magar	7 (15.22)
Darai	4 (8.70)
Brahamin	3 (6.52)
Chhetri	1 (2.17)
Kumal	1 (2.17)
Mandai	1 (2.17)
Pariyar	1 (2.17)
Sahi	1 (2.17)
Shah	1 (2.17)

Among 46 children with high serum ferritin level, 20 (43.48%) were on regular chelation therapy (Table 4).

**Table 4:** Chelation therapy (n= 46).

Chelation therapy	n (%)
No	23 (50)
Regular	20 (43.48)
Irregular	3 (6.52)

## DISCUSSION

The prevalence of high serum ferritin levels in our study was found to be 46 (88.6%) which is higher than a similar study conducted in India where the prevalence was 87.50%.^6^ In our study, the majority of the children 34 (73.91%) were found with serum ferritin levels between 1000 to 2500 ng/ml whereas 12 (26.09%) had more than 2500 ng/ml. A similar result was observed in a study where the majority of the cases had ferritin levels of more than 1000 ng/ml and only 12.5% of cases had less than 1000 ng/ml.^6^ Those who were on regular chelation therapy had low ferritin levels.

In the present study, male predominance was seen, with a male: female ratio of 1.4:1. Similarly, a study from India in 2016 had male predominance with thalassemia prevalence of 53.3% in males and 46.6% in females.^7^ Also, a study from Tunisia had a higher percentage of thalassemia in males (55.4%).^8^

In our study, the mean age of the children was 8.66±4.064 years whereas 47.8% of children were between the ages of 10 to 14 years. A similar result was seen in a study conducted in Pakistan where the mean age was 10.8±4.5 years and 46.5% of children were from the 10 to 14 years age group.^9^ The majority of the children were diagnosed before infancy which was similar to the other study.^2^ In the present study, the mean age was 15.58 months, with a range between 2 months to 97 months.

In the present study also 26 (56.52%) were from Tharu populations. Though thalassemia is more prevalent in the Tharu community, Magar, Darai, and Brahamin were also found to be affected; which shows screening and genetic counselling are mandatory irrespective of a particular caste. A study in 2020 found that thalassemia was more prevalent in Tharu communities.^10^

In the present study, only 20 (43.47%) children were on regular chelation therapy because of the unavailability of this facility in our centre. Unavailability and affordability of chelation therapy were one of the major reasons for iron overload and transfusion-related complications in thalassemia children which were also observed in another study.^11^

There were a few limitations of this study. It is a single-centre study with a small sample size done in the Department of Paediatrics, therefore it cannot be generalized to the whole population.

## CONCLUSIONS

The prevalence of high serum ferritin levels among blood transfused thalassemic patients admitted to the Department of Paediatrics in a tertiary care centre was found to be higher than in other studies done in similar settings. So those on multiple transfusion therapy need to be monitored for serum ferritin regularly so that early chelation therapy can be started to prevent iron overload and its complication. Community education, genetic counselling, and availability of transfusion, as well as early chelation therapy, will make a healthy life for suffering children.
